# Hydroxychloroquine enhances efferocytosis and modulates inflammation via MerTK/Gas6 signaling in a pristane-induced lupus mouse model

**DOI:** 10.3389/fimmu.2025.1524315

**Published:** 2025-06-16

**Authors:** Shin-Yi Liu, Yung-Ju Yeh, Ting-Yin Xue, Fei-Hung Hsieh, Ya-Wun Wu, Meng-Zhen Wu, Wei-Jing Li, Jun-Chieh J. Tsay, Shu-Yao Tsai, Chung-Ming Huang, Hen-Hong Chang, Hui-Chen Chen, Chun-Ping Lin, Gregory J. Tsay

**Affiliations:** ^1^ Department of Biomedical Imaging and Radiological Science, China Medical University, Taichung, Taiwan; ^2^ Graduate Institute of Biomedical Sciences, China Medical University, Taichung, Taiwan; ^3^ Research and Development Center for Immunology, China Medical University, Taichung, Taiwan; ^4^ Division of Immunology and Rheumatology, Department of Internal Medicine, China Medical University Hospital, Taichung, Taiwan; ^5^ Division of Pulmonary and Critical Care Medicine, New York University School of Medicine, New York, NY, United States; ^6^ Division of Pulmonary and Critical Care Medicine, Veterans Administration (VA) New York Harbor Healthcare System, New York, NY, United States; ^7^ Department of Food Nutrition and Health Biotechnology, Asia University, Taichung, Taiwan; ^8^ College of Medicine, China Medical University, Taichung, Taiwan; ^9^ Department of Chinese Medicine, China Medical University Hospital, Taichung, Taiwan; ^10^ Graduate Institute of Integrated Medicine, Graduate Institute of Acupuncture Science, College of Chinese Medicine, China Medical University, Taichung, Taiwan; ^11^ Department of Microbiology and Immunology, School of Medicine, China Medical University, Taichung, Taiwan

**Keywords:** HCQ, efferocytosis, MERTK, inflammation, SLE

## Abstract

**Background:**

Hydroxychloroquine (HCQ) is a frontline treatment for autoimmune diseases, including rheumatoid arthritis, Sjogren’s syndrome, and systemic lupus erythematosus (SLE), due to its potent immunomodulatory properties. Efferocytosis, a crucial process for tissue homeostasis by transmitting immune-suppressive signals, is frequently impaired in SLE. We hypothesized HCQ enhances efferocytosis and mediates anti-inflammatory effects.

**Methods:**

A pristane-induced lupus (PIL) mouse model was used to assess the preventive efficacy of HCQ by measuring inflammatory cytokine levels, autoantibody titers, and lupus nephritis severity. Efferocytosis in HCQ-treated macrophages was quantified following co-incubation with apoptotic cells and the expression levels of TAM family receptors post-HCQ stimulation were analyzed *in vitro* and *in vivo*. The role of MerTK on HCQ-modulated inflammation was revealed by MerTK inhibitor UNC2025.

**Results:**

Long-term HCQ treatment in PIL mice significantly reduced disease activity. HCQ treatment enhanced efferocytosis in RAW264.7 cells, while peritoneal macrophages from HCQ-treated mice showed increased efferocytotic capacity compare to PIL mice. Additionally, HCQ upregulated the expression of the TAM receptor MerTK and Gas6 on macrophages, restoring MerTK levels suppressed by pristane in the spleen of PIL mice. Inhibition of MerTK signaling by UNC2025 mitigated HCQ-mediated enhancements in efferocytosis and reversed the reduction in inflammatory mediators including IL-6 and IFN-α. HCQ-induced anti-inflammatory markers, such as PPARγ, LXR, and IL-10, were also alleviated upon MerTK blockade.

**Conclusion:**

This study provides robust *in vitro* and *in vivo* evidence that HCQ promotes macrophage efferocytosis and anti-inflammatory reprogramming via MerTK/Gas6 signaling, offering insights into potential therapeutic mechanisms in SLE management.

## 1 Introduction

Systemic lupus erythematosus (SLE) is a complex autoimmune disease characterized by dysregulated immune responses and multi-organ inflammation (1). Aberrant activations both in innate and adaptive immune compartments contribute to the pathogenesis of SLE (2, 3). Treatment for SLE is generally based on nonspecific immunosuppression, including corticosteroids, methotrexate, mycophenolate mofetil, and cyclophosphamide. Biological therapies targeting B cells or interferons are emerging as effective treatment options (4). However, the complete long-term remissions after these medications are rare (5).

Hydroxychloroquine (HCQ) has proved to be effective for the treatment of many autoimmune diseases including rheumatoid arthritis, Sjøgren’s syndrome, systemic lupus erythematosus (SLE) with lower incidence of adverse reactions than chloroquine (CQ) (6, 7). The use of HCQ can decrease lupus activity, especially for the symptoms of dermatological complications and arthritis (8–10). The mechanisms of therapeutic benefit of HCQ in autoimmune diseases have been reported to inhibit different sets of cellular functions, including autophagy, cytokine signaling, NADPH oxidase signaling, calcium mobilization, and Golgi trafficking (11, 12). HCQ-mediated inhibition of autophagy is resulted from the increase of the lysosomal pH with a consequent defect of endolysosomal activities, leading to impaired antigen presentation and Toll-like receptor (TLR) function (13–15). Other effects independent of endolysosomal function and autophagosome-lysosome fusion were also declared for HCQ. It has been stated that HCQ exhibits the T-cell-intrinsic immunomodulatory properties through interfering mitochondrial antioxidant system essential for the clearance of ROS generated from TCR crosslinking, resulting in the reduction of CD4^+^ T cell proliferation (16). Moreover, HCQ can block the cyclic GMP–AMP synthase (cGAS)–stimulator of interferon genes (STING) pathway through direct interaction with nucleic acids and inhibiting interferon beta (IFN-β) production (17–19). However, the early mechanism of action of HCQ for the treatment of SLE remain to be elucidated.

Macrophages play a key role to clear apoptotic cells by phagocytosis, a process termed efferocytosis (20). Efferocytosis is critical for the maintenance of tissue integrity, immune homeostasis and preventing autoimmunity (21, 22). Several receptors on macrophages are responsible for recognizing “eat-me” signals displayed by apoptotic cells, such as phosphatidylserine, oxidized low-density lipoproteins, and modified proteins (23, 24). These receptors include PS recognition receptors (TIM-4, BAI1, and stabilin-2), scavenger receptors (CD36, SR-A, and SR-BI), TAM Receptors (Tyro3, Axl, and MerTK) and other receptors (CD14, CD68, and CD91) (25, 26). Digestion of apoptotic cells by macrophages leads to an overload of cholesterol from degraded apoptotic cells and stimulates nuclear receptors including peroxisome proliferator-activated receptor gamma (PPARγ) and liver X receptor (LXR) to produce anti-inflammatory cytokines and to drive M2-like polarization (27–29). Apoptotic neutrophils, which are abundant during inflammatory responses, release pro-inflammatory molecules and autoantigens if not promptly cleared. In SLE, impaired efferocytosis has been identified as a key pathological feature in both mice and humans (30, 31). This failure to effectively clear apoptotic cells leads to the accumulation of cellular debris, which serves as a source of autoantigens. These autoantigens stimulate the production of autoantibodies, driving the formation of immune complexes that deposit in tissues, causing inflammation, organ damage, and characteristic lupus manifestations such as nephritis, arthritis, and skin lesions (32, 33). To investigate the early actions of HCQ in SLE, we examined its impact on efferocytosis and TAM receptor regulation in a pristane-induced lupus (PIL) mouse model. We hypothesize that HCQ treatment enhances the efferocytic capacity of macrophages, subsequently promoting anti-inflammatory responses. Understanding the mechanisms by which HCQ modulates efferocytosis may provide insights for the development of novel therapeutic strategies for the treatment of SLE.

## 2 Materials and methods

### 2.1 Cell culture

RAW264.7 and EL4 cells were procured from the Bioresource Collection and Research Center (Hsinchu, Taiwan). RAW264.7 cells were maintained in Dulbecco’s Modified Eagle Medium (DMEM; Gibco, Grand Island, NY) supplemented with 10% fetal bovine serum (FBS; HyClone, Logan, UT, USA) and EL4 cells were cultured in DMEM containing 10% horse serum (HyClone, Logan, UT, USA) supplemented with 2 mM glutamine, 100 U/mL penicillin, and 100 μg/mL streptomycin. Unless otherwise specified, all chemicals were obtained from Sigma-Aldrich (St. Louis, MO, USA).

### 2.2 Cell viability assay

RAW264.7 cells were treated with varying concentrations of hydroxychloroquine (HCQ) or chloroquine (CQ) (1.25-40 μM; Cayman Chemical, Ann Arbor, Michigan) for 24 hours. Cell viability was determined via the MTT assay (3-(4,5-dimethylthiazol-2-yl)-2,5-diphenyltetrazolium bromide; BioVision, Milpitas, CA, USA) following the manufacturer’s protocol.

### 2.3 Pristane-induced lupus mouse model

Twenty-one 6-week-old female BALB/c mice were obtained from LASCO (Taipei, Taiwan) and allocated into three groups: the normal group (Group 1, n=7), the PIL group induced with pristane (Group 2, n=7), and the HCQ-treated PIL group (Group 3, n=7). The Institutional Animal Care and Use Committee of China Medical University Hospital, Taichung, Taiwan, approved the study protocol (CMUIACUC-2018-282-1, June 2018-June 2019), and the experiments adhered to ARRIVE guidelines. PIL was induced via a single intraperitoneal injection of pristane (0.5 mL; Sigma Chemical, St. Louis, MO, USA) on day 0. HCQ was administered via oral gavage (3 mg/kg/day; ACROS, Geel, Belgium), starting on day 1 and continued for 180 consecutive days. Peritoneal lavage and plasma were collected at 1, 3, and 6 months post-induction for the analysis of lupus-related markers, including autoantibody production, inflammatory cytokines, and proteinuria. Mice were sacrificed at 6 months to evaluate lupus nephritis through immune complex deposition in the kidneys. In the short-term treatment study, mice received HCQ (30 mg/kg/day) by oral gavage for seven consecutive days.

### 2.4 Immunohistochemistry

Paraffin-embedded kidney tissues from mice were sectioned into 5-µm serial slices, which were dried overnight at 60°C. The sections were deparaffinized in purified xylene (Leica) for two 10-minute immersions, followed by rehydration through a graded ethanol series (100%, 95%, 80%, and 70%) for 5 minutes each, with a final 5-minute wash in tap water. Antigen retrieval was achieved using a heat-induced epitope retrieval method with Citrate Antigen Retrieval Solution (Scytek) for 30 minutes. The slides were then washed in TBST (Scytek) for 5 minutes, blocked for endogenous peroxidase activity with Peroxide Block (ScyTek) for 10 minutes, and washed again twice with TBST. Non-specific binding was minimized by incubating slides with Protein Block (Leica Novolink) for 5 minutes, followed by two additional TBST washes. A goat anti-mouse IgG (H+L)-HRP antibody was diluted 1:200 in antibody dilution buffer (Ventana) and applied to the sections for 30 minutes, then washed twice with TBST. The sections were developed with DAB (Leica Novolink) for 5 minutes and rinsed with tap water. Counterstaining was performed with Hematoxylin Gill II (Leica) for 2 minutes, followed by another 5-minute wash in tap water. Finally, slides were dehydrated through two 5-minute immersions in 100% alcohol, cleared in xylene, and mounted with Micromount medium (Leica). The immunostained areas of IgG in kidney tissues were captured using an Axio Observer A1 light microscope (ZEISS, Germany) and subsequently analyzed with ImageJ software (NIH, Bethesda, MD, USA). Glomerular IgG staining was evaluated and graded on a scale from 0 (no staining) to 4 (strong, global mesangial staining) as previously described (34). For quantification, all IgG-positive cells within each glomerular tuft were counted and normalized to the total number of glomeruli present in the biopsy. A minimum of 25 glomeruli were assessed per sample to ensure robust quantification.

### 2.5 Flow cytometry

Prior to staining with cell surface markers, cells were stained using the Zombie Aqua fixable viability kit (BioLegend, San Diego, CA, USA) according to the manufacturer’s instructions. Surface expression of target proteins was evaluated by staining with fluorochrome-conjugated antibodies (BioLegend, San Diego, CA), including anti-F4/80-Alexa488 (clone BM8), anti-CD19-PE (clone 6D5), anti-MerTK-PE/Cy7 (clone 2B10C42), and anti-CD11b-BV421 (clone M1/70) for 20 minutes at 4°C. After washing, the cells were analyzed with a BD FACSCelesta flow cytometer (BD Biosciences, Franklin Lakes, NJ, USA) and data were processed using FlowJo software (Tree Star, Ashland, OR, USA). Apoptosis was performed by using ApoScreen Annexin V Apoptosis Kit (Southern Biotech, Birmingham, AL, USA) according to the manufacturer’s protocol.

### 2.6 *In vitro* efferocytosis assay

RAW264.7 cells were stained with 5 μM carboxyfluorescein succinimidyl ester (CFSE; eBioscience, Santa Clara, CA, USA) for 20 minutes at 37°C and subsequently treated with HCQ for 24 hours. EL4 cells were labeled with 0.5 μM MitoTracker Deep Red FM (Thermo Fisher Scientific, Waltham, MA, USA) for 30 minutes at 37°C, followed by UVB exposure at 500 J/m^2^ to induce apoptosis (34). One day post-exposure, the apoptotic EL4 cells were co-cultured with RAW264.7 cells at a 10:1 ratio for 30 minutes to 2 hours at 37°C. After washing with PBS, the cells were stained with Zombie Aqua live-dead dye (BioLegend, San Diego, CA, USA) and analyzed using the BD FACSCelesta flow cytometer. Efferocytosis was quantified as the percentage of macrophages engulfing apoptotic cells (Deep Red^+^/CSFE^+^) out of the total live macrophages (Zombie^-^/CSFE^+^) (35). For pHrodo staining, apoptotic EL4 cells in serum-free RPMI 1640 medium were labeled by pHrodo red succinimidyl ester (Thermo Fisher Scientific, Waltham, MA, USA) at a final concentration of 1 μM and incubated with CSFE-stained RAW264.7 cells for 1 hour at 37°C in the dark. After labeling, cells were washed three times with PBS and resuspended in complete culture medium and analyzed using the BD FACSCelesta flow cytometer.

### 2.7 Ex vivo efferocytosis assay

Thymocytes were harvested from BALB/c mouse thymi and induced to undergo apoptosis via serum starvation for 24 hours (36). Peritoneal macrophages were isolated from untreated, PIL, and PIL+HCQ mice on day 7 post-PIL. After a 4-hour adherence period, the macrophages were co-incubated with MitoTracker Deep Red-labeled apoptotic thymocytes at a 10:1 ratio for 30 minutes at 37°C. Efferocytosis was quantified as the percentage of macrophages engulfing apoptotic thymocytes (Deep Red^+^/F4/80^+^) relative to total live macrophages (Zombie^-^/F4/80^+^) using the BD FACSCelesta flow cytometer.

### 2.8 Immunofluorescence staining

After engulfing apoptotic EL4 cells (Deep Red^+^) for 30 min, RAW264.7 cells (CSFE^+^) were washed three times with PBS and analyzed using an Axio Observer A1 inverted fluorescence microscope (ZEISS, Germany). Effercytosis is defined by the counts of Deep Red^+^ cells within CSFE-positive region using ImageJ software (NIH, Bethesda, MD, USA). Acquired images were converted to 8-bit grayscale and processed using the “Threshold” function to segment positive signals from the background. A region of interest (ROI) was selected according to CFSE-green image using the selection tool and subsequently applied to the corresponding red-channel image to define the analysis area. The counts of positively Deep Red-stained cells within the defined ROI were automatically detected using the Analyze Particles function and then exported for statistical analysis. CFSE-green signals were collected using an Argon ion laser (488nm) and 525 bandpass filter, while Deep Red were detected with Cy5 filter at 40x magnification.

### 2.9 Enzyme-linked immunosorbent assay

Levels of inflammatory cytokines IL-6, TNF-α, IFN-α, IFN-γ, IL-17A, and IL-10 were measured in peritoneal lavage and plasma samples using ELISA kits (BioLegend, San Diego, CA, USA) following the manufacturer’s instructions. Anti-nuclear antibody (ANA) and anti-double-stranded DNA (anti-dsDNA) antibodies were detected using kits from Alpha Diagnostic International (San Antonio, TX, USA) and Chondrex (Redmond, WA, USA), respectively.

### 2.10 Real-time quantitative polymerase chain reaction

Total RNA was extracted using TRIzol (Invitrogen, Carlsbad, CA, USA) and reverse-transcribed into complementary DNA (cDNA) using iScript gDNA Clear cDNA Synthesis Kit with T100 Thermal Cycler (Bio-Rad, Hercules, CA, USA). Q-PCR was conducted with IQ SYBR Green Supermix (Bio-Rad, Hercules, CA, USA) on the CFX Connect Real-Time PCR Detection System (Bio-Rad, Hercules, CA, USA) using the following parameters: initial denaturation at 95°C for 2 minutes, followed by 40 cycles of 95°C for 5 seconds, 60°C for 30 seconds, 72°C for 20 seconds. Primer sequences are listed in **Table 1**, with GAPDH used as the reference gene. Gene expression levels were quantified using the comparative ΔΔCt method, and RAW264.7 cells without EL4 cell co-incubation were designated as the calibrator group.

**Table 1:** Sequences of primer pairs used in real-time PCR.

Target	Forward Sequences	Reverse Sequences
Mertk	5’-CGCTTCCTTCAGCATAACCA-3’	5’-TTCATGCTCTCAGGCTGCTT-3’
Axl	5’-GGTCAGCCAGCTCAGAATCAC-3’	5’-TCCTCCAGGAAGTAAGGCAAG-3’
Gas6	5’-TACAGCCTGGACTACATGCG-3’	5’-GGATGTGAGCCACGACTTCT-3’
Pros	5’-ACAACTTGCCGTCTTGGACA-3	5’-GGCACTGAATGGAACATCTGG-3’
Ifna	5’-GGACTTTGGATTCCCGCAGGAGAAG-3’	5’-GCTGCATCAGACAGCCTTGCAGGTC-3’
Il-6	5’-CCAGTTTGGTAGCATCCATCATTTC-3’	5’-CCACTTCACAAGTCGGAGGCTTA-3’
Il-10	5’-CAGTACAGCCGGGAAGACAA-3’	5’-AAATCGATGACAGCGCCTCAG-3’
Gapdh	5’-TGCAAAGTGGAGATTGTTGCC-3’	5’-AAGAATGGTGATGGGCTTCCCG -3’

### 2.11 Western blot analysis

Frozen tissue samples (100 mg) were homogenized in grinding ball with two cycles of 60Hz of oscillation frequency for 30 sec using tissue grinders (High-throughput Tissue Grinders NANBEI, NB-48P). Protein lysates were lysed in RIPA buffer (Chemicon, Millipore, USA) and quantified by Assay Dye Reagent Concentrate (Bio-Rad #5000006). Proteins were separated by SDS-PAGE, transferred onto polyvinylidene difluoride (PVDF) membranes (Immobilon, Millipore, Eschborn, Germany) using Wet/Tank Blotting Systems (Bio-Rad, Hercules, CA, USA), and blocked with 3% BSA in TBS (pH 7.4) for 1 hour at room temperature. The membranes were incubated overnight at 4°C with primary antibodies against MerTK (AF591, R&D), p-MerTK (p186-749, PhosphoSolutions), Gas6 (PA5-72882, Thermo Fisher Scientific), p62 (A11250, ABclonal), LC3B (E-AB-70053, Elabscience), PPARγ (E-AB-60059, Elabscience), LXR (A04523-2, Boster), and actin (3700, Cell Signaling). After washing, membranes were probed with secondary antibodies (Elabscience), and detection was performed using ECL Plus reagent (PerkinElmer, MA, USA) on a BOX Chemi XRQ imaging system (Syngene, Cambridge, UK). Protein intensity was normalized to actin in each lane first and then calculated fold change relative to compared to the control condition.

### 2.12 Statistical analysis

Statistical analyses were conducted using GraphPad Prism software. Data are expressed as mean ± standard error of the mean (SEM) unless otherwise stated. The Shapiro-Wilk test was used to assess normality of data distribution. For comparisons between two independent groups, an unpaired Student’s t-test was performed for normally distributed data, whereas the Welch’s t-test was applied when homogeneity of variances was not met. For non-normally distributed data, the Mann-Whitney U test was utilized. For multiple-group comparisons, Welch’s ANOVA, followed by an appropriate *post hoc* test, was applied when variances were unequal. For non-normally distributed data, the Kruskal-Wallis test, followed by Dunn’s *post hoc* test, was used for multiple comparisons. Statistical significance was defined as p < 0.05.

## 3 Results

### 3.1 HCQ reduces inflammation and lupus nephritis in a lupus mouse model

**Figure 1:**
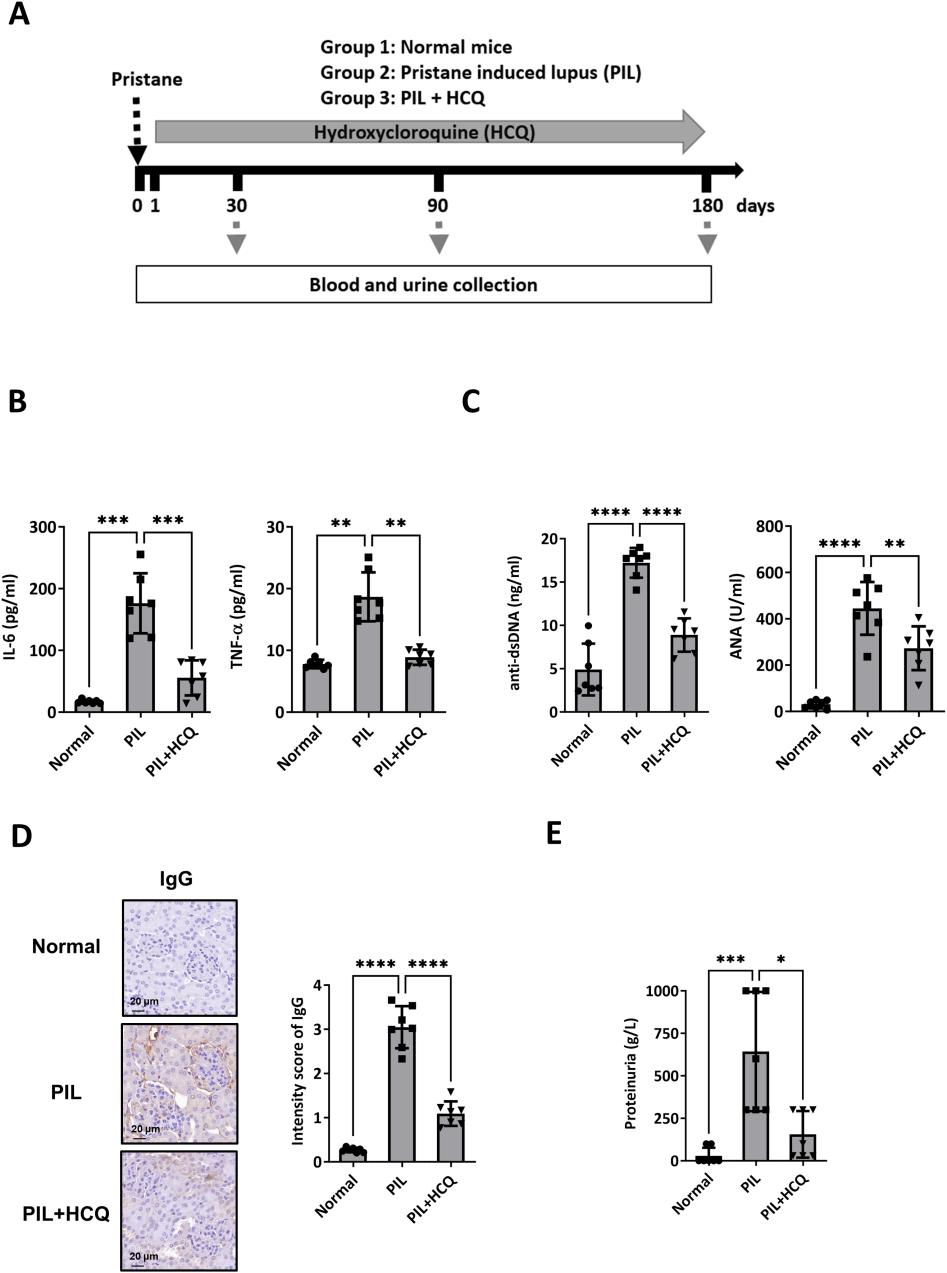
Therapeutic efficacy of HCQ in a lupus model mouse. **(A)** Female BALB/c mice were divided into three groups: Group 1: normal mice (normal group, n=7), Group 2: pristane-induced lupus mice (PIL group, n=7), and Group 3: PIL mice given HCQ (PIL+HCQ group, n=7). Intraperitoneal injection of pristane was defined as day 0, and HCQ administration (3 mg/kg/day) began on day 1 post-pristane injection and continued for 180 days. Serum and urine samples were collected at 30, 90, and 180 days after induction. **(B)** The levels of IL-6 (left) and TNF-α (right) in peritoneal lavage 6 months after PIL induction in the normal, PIL, and PIL+HCQ groups by ELISA analysis (n=7). **(C)** The level of anti-dsDNA-specific IgG (left) and anti-nuclear antibody (ANA, right) in plasma from the normal, PIL, and PIL+HCQ groups was accessed after 6 months of treatment by ELISA assay (n=7). **(D)** Representative immunohistochemistry images (left) and quantitative analysis (right) of IgG deposition in kidney sections from the normal, PIL, and PIL+HCQ groups after 6 months of treatment (n=7). **(E)** Urine protein levels were measured in urine samples from the normal, PIL, and PIL+HCQ groups after 6 months of treatment (n=7). Scale bar: 20 μm. *p < 0.05; **p < 0.01; ***p < 0.001; ****p < 0.0001.

To investigate the underlying mechanism by which HCQ may slow the progression of SLE, a mouse model was established using pristane to induce lupus-like symptoms. A total of 21 female mice were randomly assigned to one of three experimental groups. Group 1 served as the control, receiving sterile saline injections (normal group, n=7); Group 2 was administered pristane to induce a lupus phenotype (PIL group, n=7); and Group 3 was comprised of pristane-induced lupus (PIL) mice treated with HCQ (PIL+HCQ group, n=7). HCQ was given orally at a dose of 3 mg/kg per day, beginning on day 1 after PIL induction, with daily treatment continuing for a period of 180 days to assess long-term effects on disease progression. To evaluate the preventive effects of HCQ, plasma and urine samples were collected at 1, 3, and 6 months post-PIL induction and analyzed for inflammatory cytokine levels, anti-double-stranded DNA (anti-dsDNA) antibody titers, and proteinuria as indicators of disease activity and progression. After 6 months, all mice were sacrificed to permit in-depth examination of immune complex deposition and lupus nephritis through histopathological and immunological analysis (**Figure 1A**). Our results revealed that HCQ treatment had a significant suppressive effect on pro-inflammatory cytokine production, including interleukin-6 (IL-6) (p=0.006) and tumor necrosis factor-alpha (TNF-α) (p=0.0012) in peritoneal lavage of PIL mice at the 6-month time point (**Figure 1B**). Additionally, HCQ significantly reduced circulating anti-dsDNA antibody (p<0.0001), anti-nuclear antibody (ANA) (p<0.0041), and total IgG levels (p=0.0003), a hallmark of lupus, at 6 months post-PIL induction (**Figure 1C**, **Supplementary Figure S1A**). Histological examination showed that HCQ treatment led to a substantial reduction in immune complex deposition within the kidneys (**Figure 1D**) and complement C3 deposition (**Supplementary Figure S1B**), which correlated with an alleviation of proteinuria (p=0.0385) when compared to untreated PIL mice (**Figure 1E**). These findings indicate that HCQ effectively mitigates inflammatory signals, especially IL-6 and TNF-α, and protects against renal damage associated with lupus nephritis.

### 3.2 HCQ enhances efferocytosis *in vitro*

**Figure 2:**
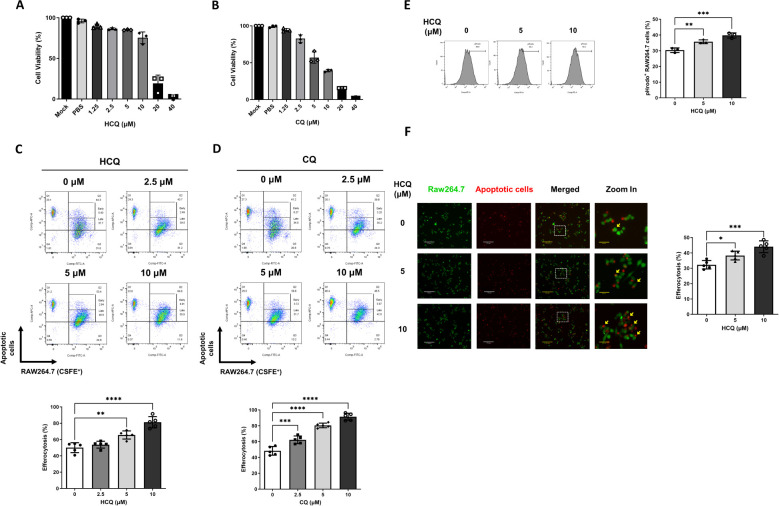
HCQ enhances efferocytosis *in vitro*. **(A, B)** The viability of RAW264.7 cells treated with varying concentrations of HCQ **(A)** and chloroquine (CQ) **(B)** was assessed using MTT assays over 24 hours (n=3). **(C, D)** Representative flow cytometry images (upper) and data plots (lower) show efferocytotic capacity of RAW264.7 cells (CFSE-labeled, green) treated with subtoxic doses of HCQ **(C)** or CQ **(D)** (2.5-10 μM) in the presence of apoptotic EL4 cells (Deep Red labeled, red) for 1 hour (n=5). **(E)** Representative flow cytometry images (left) and data plot (right) indicate the percentage of pHrodo red-positive cells within RAW264.7 cells treated with HCQ after 4 hours of co-incubation (n=3). **(F)** Representative immunofluorescent images (left) and data plot (right) display the percentage of apoptotic EL4 cells phagocytosed by HCQ-treated RAW264.7 cells after 30 minutes of co-incubation (n=5). Scale bar: 170 μm. *p < 0.05; **p < 0.01; ***p < 0.001; ****p < 0.0001.

Given the observed reduction of pro-inflammatory cytokines IL-6 and TNF-α in PIL mice treated with HCQ, we hypothesized that HCQ enhances macrophage-mediated efferocytosis, thus promoting anti-inflammatory signaling pathways. To investigate this, we used murine macrophage-like RAW264.7 cells as an *in vitro* model. Cytotoxicity assays revealed that the half-maximal inhibitory concentration (IC50) of HCQ in RAW264.7 cells after 24 hours was 14 μM, while the IC50 of CQ was notably lower at 5 μM, indicating HCQ’s relatively milder cytotoxicity profile (**Figures 2A, B**). To evaluate the effect of HCQ on macrophage efferocytosis, apoptotic cells were generated by UV irradiation of EL4 cells and subsequently prepared for co-incubation with macrophages (**Supplementary Figure S2**). RAW264.7 cells were labeled with CSFE and treated with sub-toxic concentrations of HCQ and CQ (2.5, 5, and 10 μM) for 24 hours, followed by co-incubation with apoptotic EL4 cells labeled with MitoTracker Deep Red to facilitate tracking. Efferocytosis was quantified by flow cytometry, with the efferocytic macrophages defined as the percentage of double-positive MitoTracker Deep Red and CFSE cells (Deep Red^+^/CFSE^+^) out of the live macrophages (Zombie^-^/CFSE^+^). HCQ treatment significantly enhanced macrophage efferocytosis in a dose-dependent manner, with a pronounced increase observed at 5 μM (p=0.0014). Similarly, CQ treatment showed significant efferocytic enhancement at 5 μM (p<0.0001) (**Figures 2C, D**, **Supplementary Figure S3**). We utilized pHrodo-Red, a pH-sensitive fluorescent dye that selectively fluoresces in acidified lysosomes, to label apoptotic cells and assess efferocytosis in macrophages. Following a 4-hour co-incubation period, flow cytometric analysis revealed a significant increase in the proportion of pHrodo-positive RAW264.7 cells (pHrodo^+^/CFSE^+^). Treatment with HCQ led to a marked elevation in pHrodo fluorescence (p=0.0072 at 5 μM), indicating HCQ facilitates the uptake and lysosomal processing of apoptotic cells by macrophages (**Figure 2E**). To visually confirm these observations, immunofluorescence analysis was performed, which demonstrated an increased phagocytosis of apoptotic cells to HCQ-treated macrophages compared to untreated controls (p=0.0206 at 5 μM), further supporting the efferocytic capacity induced by HCQ treatment (**Figure 2F**). These findings indicate that HCQ, within sub-toxic dose ranges, effectively enhances macrophage efferocytosis.

### 3.3 HCQ increases MerTK expression *in vitro*

**Figure 3:**
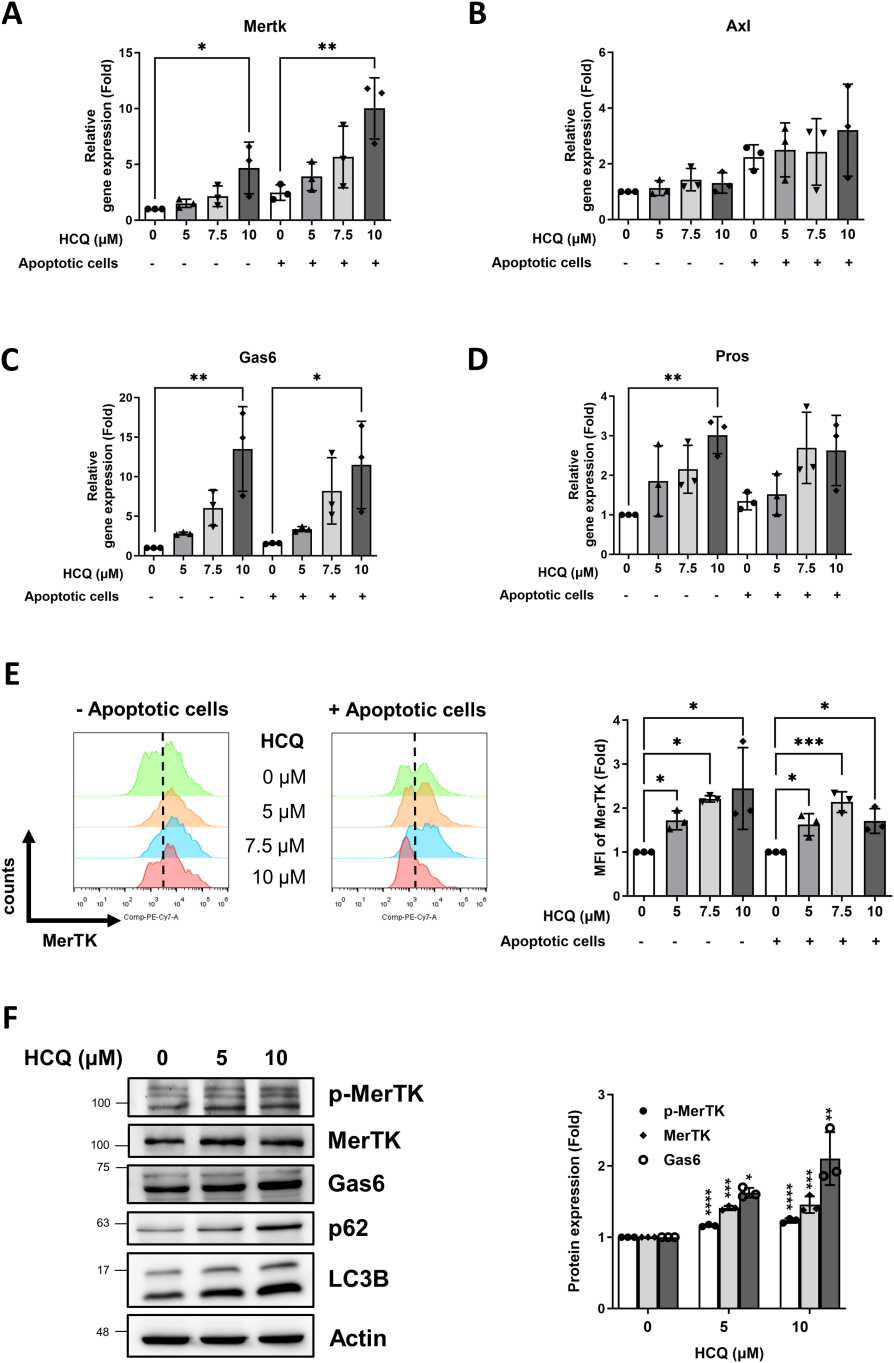
HCQ increases MerTK expression *in vitro*. **(A, B)** Gene expression of TAM receptors Mertk **(A)** and Axl **(B)** in RAW264.7 cells treated with indicated concentration of HCQ (5, 7.5, 10 μM) for 24 hours with or without co-incubation with apoptotic EL4 cells (n=3). **(C, D)** Gene expression of TAM ligands Gas6 **(C)** and Pros **(D)** in RAW264.7 cells treated with indicated concentration of HCQ (5, 7.5, 10 μM) for 24 hours with or without co-incubation with apoptotic EL4 cells (n=3). **(E)** Representative flow cytometry images (right) and data plot (left) show surface expression of MerTK on RAW264.7 cells treated with indicated concentration of HCQ (5, 7.5, 10 μM) for 24 hours with or without co-incubation with apoptotic EL4 cells (n=3). **(F)** Representative WB images (left) and data plot (right) show the protein expression of MerTK, p-MerTK, Gas6, p62, and LC3B in RAW264.7 cells treated with HCQ (5 and 10 μM) for 24 hours (n=3). MFI refers to the median fluorescence intensity. *p < 0.05; **p < 0.01; ***p < 0.001; ****p < 0.0001.

To further elucidate the mechanism by which HCQ enhances efferocytosis, we analyzed the gene expression profiles of apoptotic cell recognition receptors on macrophages, focusing on a variety of receptors essential for efferocytosis. These included Mertk, Axl, Tyro3, Tim4, Itgb3 (integrin β3), and Cd36, both in the absence and presence of apoptotic cells. Additionally, we assessed the gene expression of soluble phosphatidylserine (PtdSer)-binding proteins, namely Pros (protein S), Gas6, and Mfge8, which facilitate apoptotic cell recognition and clearance. Our findings reveal that HCQ treatment at a concentration of 10 μM significantly upregulates Mertk gene expression in RAW264 macrophages, under conditions both with (p=0.0179) and without (p=0.0055) apoptotic cell co-incubation, indicating a consistent HCQ effect on this pivotal efferocytosis receptor (**Figure 3A**). HCQ also markedly increased the expression of Gas6, a key ligand for TAM receptors, with significant upregulation observed at 10 μM both in the absence (p=0.002) and presence (p=0.0202) of apoptotic cells (**Figure 3C**). While increases in Axl and Pros expression were observed, these trends did not reach statistical significance following apoptotic cell co-incubation (**Figures 3B, D**). Other gene expressions of efferocytosis-related molecules (Tyro3, Tim4, Itgb3, Cd36, Mfge8) in macrophages were not changed after HCQ treatment. At the cell surface level, flow cytometric analysis revealed that HCQ also significantly increased the surface expression of MerTK on macrophages, observed at 5 μM(p=0.045), 7.5 μM (p=0.0346), and 10 μM (p=0.0144) in the absence of apoptotic cells (**Figure 3E**). After expose to apoptotic cells, HCQ-treated macrophages also exhibited increased surface MerTK expression. This upregulation is particularly notable, as it highlights HCQ’s ability to enhance MerTK surface availability, which is essential for efficient efferocytosis. The reduced MerTK levels in macrophages treated with 10 μM HCQ were attributed to the deterioration of death following co-incubation with apoptotic cells. Additionally, HCQ administration effectively inhibited autophagy, as evidenced by increased protein levels of p62 and LC3B, markers indicative of disrupted autophagic flux (**Figure 3F**). Notably, the levels of p-MerTK and Gas6 was elevated in a dose-dependent manner following HCQ treatment, suggesting that HCQ modulates MerTK and Gas6 proteins at both transcriptional and translational levels (**Figure 3F**). These results support the notion that HCQ upregulates MerTK and Gas6, highlighting HCQ’s role in promoting macrophage-mediated clearance of apoptotic cells through efferocytic pathways.

### 3.4 HCQ enhances efferocytosis, MerTK expression and reduces inflammation *in vivo*

**Figure 4:**
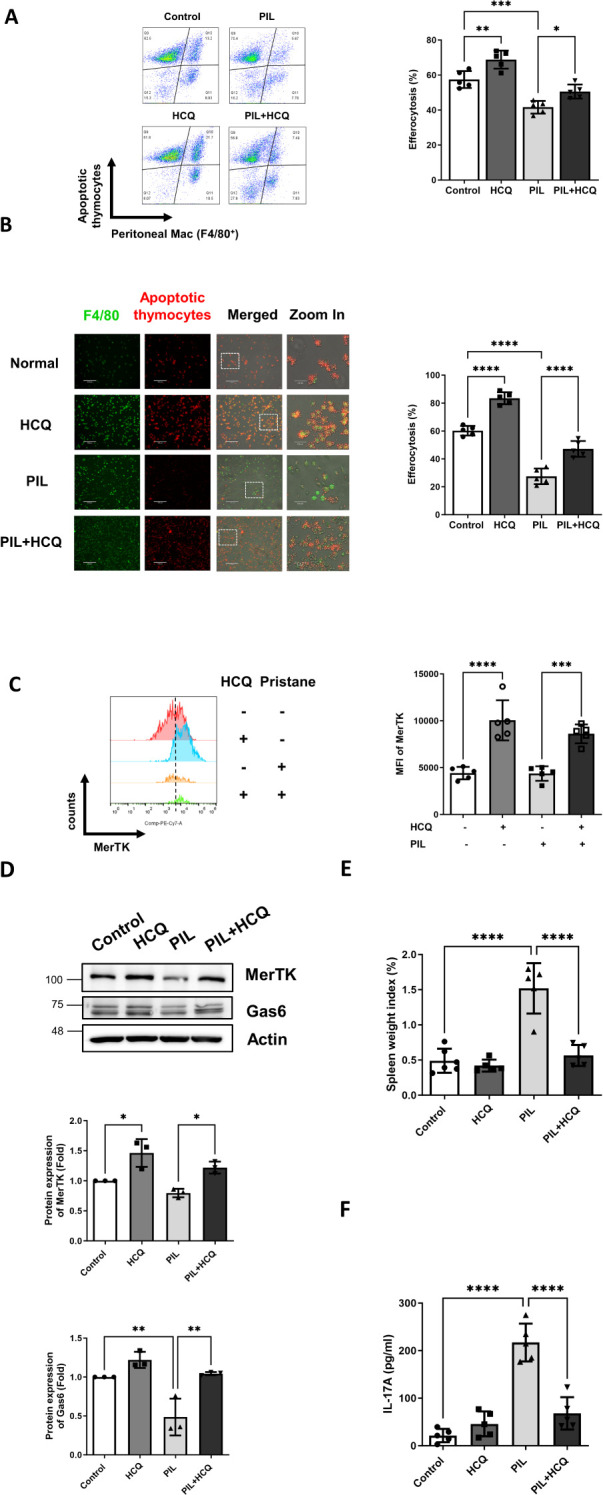
HCQ enhances efferocytosis, MerTK expression and reduces inflammation *in vivo*. **(A)** Representative flow cytometry images (left) and quantification (right) show efferocytosis of the peritoneal macrophages isolated from control, HCQ (30 mg/kg), PIL and PIL+HCQ mice after 1 week of treatment exposed to apoptotic thymocytes (Deep Red-labeled) for 30 minutes (n=5). **(B)** Representative immunofluorescence images (left) and data plot (right) display peritoneal macrophages attached to culture dishes from control, HCQ, PIL, and PIL+HCQ mice after one week of treatment, exposed to apoptotic thymocytes (Deep Red-labeled) for 30 minutes (n=5). **(C)** Representative flow cytometry images (left) and data plot (right) show the surface expression of MerTK on the peritoneal macrophages isolated from control, HCQ, PIL, and PIL+HCQ mice after 3 day of treatment (n=5). **(D)** Representative western blot images (left) and data plot (right) show the protein expression of MerTK and Gas6 in the spleen isolated from control, HCQ, PIL, PIL+HCQ mice after 1 week of treatment (n=3). **(E)** Spleen weight index from control, HCQ, PIL, and PIL+HCQ mice after 1 week of treatment (n=5). **(F)** The plasma levels of IL-17A in control, HCQ, PIL, and PIL+HCQ mice after 1 week of treatment (n=5). Scale bar: 170 μm. MFI refers to the median fluorescence intensity. *p < 0.05; **p < 0.01; ***p < 0.001; ****p < 0.0001.

The impact of HCQ on enhancing efferocytosis and MerTK expression was further examined in a PIL mouse model. Peritoneal macrophages from four experimental groups: untreated control, HCQ-treated mice (30 mg/kg daily by oral gavage), PIL-induced mice, and PIL-induced mice treated with HCQ were isolated 7 days following PIL induction. After a 30-minute co-incubation with Mitotracker Deep Red-labeled apoptotic thymocytes, efferocytotic capacity was defined as the percentage of double-positive Deep Red and F4/80 cells (Deep Red^+^/F4/80^+^) out of the live macrophages (Zombie^-^/F4/80^+^) and quantified using flow cytometry. The PIL model led to a marked reduction in macrophage efferocytosis, decreasing from a baseline of 57.5% ± 4.8% in the control group to 41.6% ± 3.6% (p=0.0002). Peritoneal macrophages isolated from HCQ-treated mice exhibited enhanced efferocytosis (68.9% ± 5.1%) and showed modest improvement in efferocytosis levels following PIL treatment, achieving 50.6% ± 4.0% (p=0.0259), suggesting a protective effect of HCQ against PIL-mediated impairment of efferocytosis (**Figure 4A**). Efferocytosis capacity of peritoneal macrophages were further confirmed by a 4-hour attachment in a culture dish and subsequently exposed to apoptotic thymocytes. Immunofluorescence staining demonstrated enhanced efferocytosis of peritoneal macrophages in the PIL+HCQ group compared to the PIL group alone (p<0.0001), indicating improved efferocytosis following HCQ treatment (**Figure 4B**). *In vivo* condition, pristane treatment resulted in a substantial reduction in the total number of peritoneal macrophages (**Supplementary Figure S4**). HCQ administration significantly increased surface MerTK expression in the peritoneal macrophage population, regardless of pristane exposure (**Figure 4C**). In the spleen, HCQ administration enhanced MerTK (p=0.0174) and Gas6 protein expression (p=0.0034) following pristane treatment (**Figure 4D**). Further analysis of apoptosis levels revealed that HCQ treatment significantly reduced the proportion of apoptotic cells in both the peritoneal lavage and spleen, suggesting modulatory effect of HCQ in eliminating apoptotic cells within these immune compartments (**Supplementary Figure S5**). Beyond cellular markers, HCQ treatment alleviated splenomegaly induced by pristane (p<0.0001), suggesting that HCQ mitigates inflammation linked to SLE pathology (**Figure 4E**). HCQ significantly reduced plasma IL-17A levels (**Figure 4F**) and Il17a gene expression in the peritoneal lavage following PIL treatment (p<0.0001) (**Supplementary Figure S6A**). In addition, HCQ markedly increased gene expression of anti-inflammatory cytokine Il10 in peritoneal macrophages post-PIL (p=0.0038) (**Supplementary Figure S6B**). By suppressing IL-17A and enhancing IL-10 production as early as one week after pristane exposure, HCQ appears to attenuate pro-inflammatory signals, potentially contributing to decreased autoantibody production. These findings support the anti-inflammatory effects of HCQ at early stage of actions *in vivo*.

### 3.5 HCQ promotes efferocytosis and anti-inflammatory signals via MerTK

**Figure 5:**
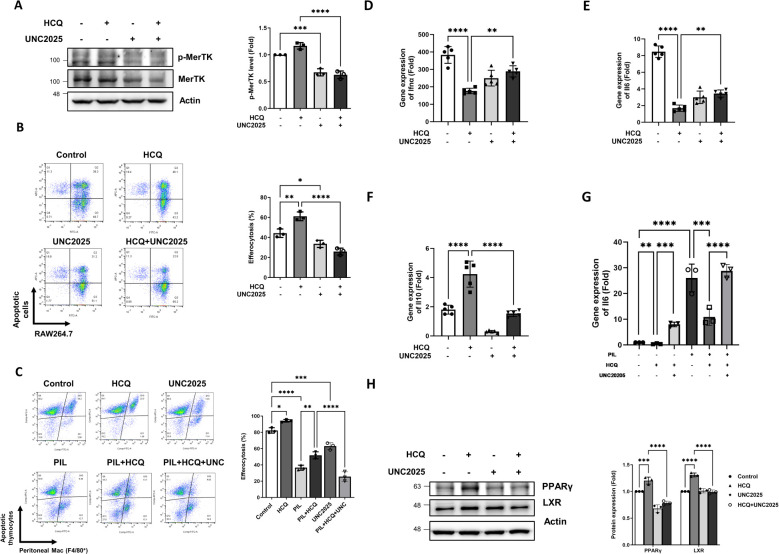
HCQ promotes efferocytosis and anti-inflammatory signals via MerTK. **(A)** Representative western blot images (left) and quantitative analysis (right) show the levels of phosphorylated MerTK and total MerTK protein in RAW264.7 cells treated with HCQ (5 μM), UNC2025 (1 μM), or HCQ+UNC2025 after co-incubation with apoptotic EL4 cells for 15 minutes. Macrophages were pretreated with UNC2025 for 1 hour prior to exposure to apoptotic cells (n=3). **(B)** Representative flow cytometry plots (left) and quantification (right) of efferocytosis in RAW264.7 cells treated with HCQ, UNC2025, or HCQ+UNC2025 following co-incubation with apoptotic EL4 cells for 1 hour (n=3). **(C)** Representative flow cytometry plots (left) and quantification (right) of efferocytosis in peritoneal macrophages isolated from normal or PIL mice treated with HCQ, UNC2025, or HCQ+UNC2025, following co-incubation with apoptotic thymocytes for 30 minutes (n=3). **(D-F)** Relative gene expression levels of Ifnα **(D)**, Il6 **(E)**, Il10 **(F)** in RAW264.7 cells treated with HCQ, UNC2025, or HCQ+UNC2025 after co-incubation with apoptotic EL4 cells for 2 hours, compared to untreated controls (n=5). **(G)** Gene expression of Il6 in peritoneal macrophages isolated from normal or PIL mice treated as indicated, following co-incubation with apoptotic thymocytes for 1 hour (n=3). **(H)** Representative western blot images (left) and quantification (right) show the expression of anti-inflammatory transcription factors PPARγ and LXR in RAW264.7 cells treated with HCQ, UNC2025, or HCQ+UNC2025 after co-incubation with apoptotic EL4 cells for 2 hours (n=3). *p < 0.05; **p < 0.01; ***p < 0.001; ****p < 0.0001.

To elucidate the role of MerTK in HCQ-enhanced efferocytosis, both RAW264.7 cells and peritoneal macrophages were treated with the specific MerTK inhibitor, UNC2025. Western blot analysis verified that UNC2025 effectively suppressed MerTK phosphorylation (**Figure 5A**), which corresponded with a significant reduction in HCQ-mediated increase in efferocytosis in RAW264.7 cells (p<0.0001) (**Figure 5B**). Similarly, peritoneal macrophages isolated from PIL mice exhibited impaired efferocytosis capacity, and the enhancement of efferocytosis by HCQ was attenuated upon MerTK inhibition (**Figure 5C**). After co-incubation with apoptotic cells, gene expressions of pro-inflammatory cytokines Ifnα and Il6 were upregulated in macrophages compared to untreated controls. HCQ treatment significantly reduced the expression of both Ifnα and Il6 (p<0.0001), an effect that was reversed after MerTK inhibition (p=0.0011 and p=0.001, respectively) (**Figures 5D, E**). In the PIL mice model, peritoneal macrophages exposed to apoptotic cells exhibited significantly increased Il6 expression, which was suppressed by HCQ treatment. This HCQ-mediated reduction in Il6 expression was reversed by MerTK inhibition with UNC2025 in both normal and PIL mice (p=0.0001 and p<0.0001, respectively) (**Figure 5G**). In contrast, exposure to apoptotic cells led to increased Il10 expression in macrophages, and this response was further enhanced by HCQ treatment (**Figure 5F**, **Supplementary Figure S6B**). Inhibition of MerTK signaling with UNC2025 significantly attenuated Il10 expression, and HCQ-induced Il10 expression was restored by UNC2025, indicating that Il10 induction is dependent on MerTK activity (**Figure 5F**). Additionally, HCQ treatment upregulated the protein expression of anti-inflammatory transcription factors, PPARγ and LXR, which are key regulators of macrophage-mediated immune modulation. Treatment of UNC2025 significantly suppressed HCQ-induced PPARγ and LXR following exposure to apoptotic cells (p<0.0001) (**Figure 5H**). These findings demonstrate that HCQ enhances efferocytosis and anti-inflammatory responses via MerTK activation, promoting IL-10 upregulation and IL-6 downregulation in macrophages.

## 4 Discussion

HCQ is widely used to treat various autoimmune diseases, including SLE, rheumatoid arthritis, and Sjögren’s syndrome, owing to its anti-inflammatory properties with infrequent serious adverse effects. The therapeutic mechanism of HCQ involves multiple pathways that modulate immune responses, including DNA/RNA binding, inhibition of toll-like receptor signaling, pH elevation in acidic compartments, reduced pro-inflammatory cytokine production, and decreased antigen presentation (37–39). This study investigates the effects of HCQ on macrophage function, providing both *in vitro* and *in vivo* evidence that HCQ enhances efferocytosis through the induction of the TAM receptor MerTK.

HCQ is known to inhibit several immune pathways, including autophagy and receptor signaling, primarily by deacidifying lysosomes and endosomes. High doses of HCQ (25-60 µM) have been reported to induce DNA damage, increased mitochondrial reactive oxygen species, endoplasmic reticulum stress, and apoptosis (40, 41). However, in this study, we used subtoxic concentrations of HCQ (2.5-10 µM) and applied live-dead staining to ensure the exclusion of dead macrophages from further analysis. Treatment with HCQ significantly increased macrophage efferocytosis and elevated the expression of Gas6 and MerTK in a dose-dependent manner. Upon binding of Gas6 to the extracellular domain of MerTK, dimerization and autophosphorylation of MerTK occur, triggering downstream signaling pathways responsible for apoptotic cell clearance, cell proliferation, and anti-inflammatory responses (42). Activation of MerTK by Gas6 inhibits the production of pro-inflammatory cytokines, such as IFN-α and IL-6, contributing to inflammation resolution (43). Inhibition of Gas6 in macrophages has been shown to attenuate autophagy and reduce the production of inflammatory cytokines (44). Conversely, Gas6 treatment induces MerTK signaling, activates autophagy, and reduces NLRP3 inflammasome activity in microglia (45). It is plausible that HCQ inhibits autophagy, promoting Gas6 expression by shifting cellular balance toward survival mechanisms and away from degradation, ultimately reducing inflammation. Whether HCQ directly increases MerTK expression via Gas6 induction requires further investigation.

Dysregulated efferocytosis has been implicated in the pathogenesis of autoimmune diseases (30). Impaired efferocytosis leads to the incomplete clearance of apoptotic cells, causing secondary necrosis and the release of damage-associated molecular patterns (DAMPs), which perpetuate inflammation. In the pristine-induced lupus (PIL) mouse model, we focused on an early stage, during which a marked accumulation of apoptotic cells were observed in the peritoneal lavage by day 2 post-pristane injection. This accumulation was significantly reduced following HCQ treatment (**Supplementary Figure S5**). By day 7, the loss of peritoneal macrophages induced by pristane was not immediately restored by HCQ treatment; however, PIL-induced inflammatory monocytes (Ly6C^++^/F4/80^med^) were significantly decreased by HCQ (**Supplementary Figure S4**). Notably, HCQ treatment alone led to a significant increase in the number of small peritoneal macrophages (**Supplementary Figure S4**) and enhanced surface expression of MerTK on peritoneal macrophages (**Figure 4C**). In addition, HCQ treatment significantly attenuated splenomegaly observed in PIL mice, accompanied by increased expression of MerTK and Gas6 in the spleen, suggesting that HCQ may promote restoration of efficient efferocytosis systemically. Given that HCQ is a chronic immunomodulatory agent used in the management of lupus, it is plausible that its effects on efferocytosis may become more pronounced at later stages of disease progression. Further studies are warranted to delineate the temporal dynamics of HCQ-mediated regulation of efferocytosis *in vivo*.

Autophagy plays a critical role in immune cell activation and pro-inflammatory cytokine release, and pristane treatment has been shown to induce autophagy in macrophages (46, 47). A hallmark of pristane-induced lupus is robust type I interferon production, and enhanced autophagy in plasmacytoid dendritic cells (pDCs) may amplify type I interferon responses, thereby activating autoreactive B and T cells (48). Our findings demonstrate that HCQ inhibits autophagy and reduces Ifnα gene expression in macrophages during efferocytosis *in vitro*. Blocking MerTK signaling reversed HCQ-mediated suppression of autophagy and Ifnα expression. In addition to regulating autophagy, HCQ modulates anti-inflammatory responses through MerTK activation in macrophages. In PIL mice, HCQ treatment significantly suppressed IL-17A production following pristane stimulation, consistent with previous studies (49, 50). IL-17A, a pro-inflammatory cytokine produced by Th17 cells, innate lymphoid cells, and γδ T cells, is an early inflammatory response induced by pristine in the plasma. Notably, Th17 cells from SLE patients with elevated type I IFN activity exhibit increased IL-17A expression (51). Emerging evidence supports a critical role for IL-17-driven pathways in SLE pathogenesis, distinct from the previously emphasized type I IFN response. Elevated IL-17-driven inflammation has been shown to promote autoreactive B-cell responses, leading to autoantibody production and the development of lupus-like features. Moreover, type I IFN and IL-17 responses can coexist, synergistically driving inflammation by recruiting and activating myeloid cells, such as neutrophils and antigen-presenting cells (52). However, the precise mechanism by which HCQ reduces IL-17A production in T cells remains unclear. Further *in vivo* investigation is warranted to elucidate these interrelated pathways between IL-17A and IFN-α and to better understand HCQ’s role in modulating inflammatory responses in SLE. Other pro-inflammatory cytokine genes, including Il1, Il6, Tnfα, and Ifnβ, are suppressed by HCQ in macrophages in response to lipopolysaccharide (LPS) stimulation (53). Our data suggest that HCQ-mediated inhibition of Ifnα and Il6 was dependent on MerTK signaling. During efferocytosis, macrophages release anti-inflammatory cytokines such as IL-10 and TGF-β to promote inflammation resolution. However, IL-10 can enhance autoantibody production in SLE patients (54). Our findings show that HCQ induces Il10 expression in macrophages *in vitro*, which is impaired by MerTK inhibition.

Our study has several limitations. Firstly, human macrophages were not used to examine the effects of HCQ on efferocytosis and cytokine profiles. Secondly, peritoneal macrophages were treated with a MerTK inhibitor ex vivo. Finally, MerTK inhibition was performed pharmacologically, and genetic depletion of MerTK in macrophages may provide more specific conclusions, though compensatory effects could arise from long-term knockout models.

In conclusion, HCQ treatment appears to be a beneficial approach in SLE by enhancing macrophage efferocytosis and promoting anti-inflammatory responses. This study reveals that HCQ exerts its immunomodulatory effects in SLE primarily by upregulating the MerTK on macrophages, thereby boosting efferocytosis. HCQ reduces pro-inflammatory cytokines and induces expression of anti-inflammatory mediators, further underscores the therapeutic advantage of autophagy inhibition in reprogramming macrophages towards a tissue-protective phenotype in SLE. Drugs like HCQ that promote efferocytosis may have significant therapeutic potential for lupus and other autoimmune diseases.

## Data Availability

The raw data supporting the conclusions of this article will be made available by the authors, without undue reservation.
