# Supplementary figures and images for: Hydroxychloroquine enhances efferocytosis and modulates inflammation via MerTK/Gas6 signaling in a pristane-induced lupus mouse model
Source: Front Immunol. 2025 Jun 16;16:1524315. doi: 10.3389/fimmu.2025.1524315 (PMC12206748; doi:10.3389/fimmu.2025.1524315)

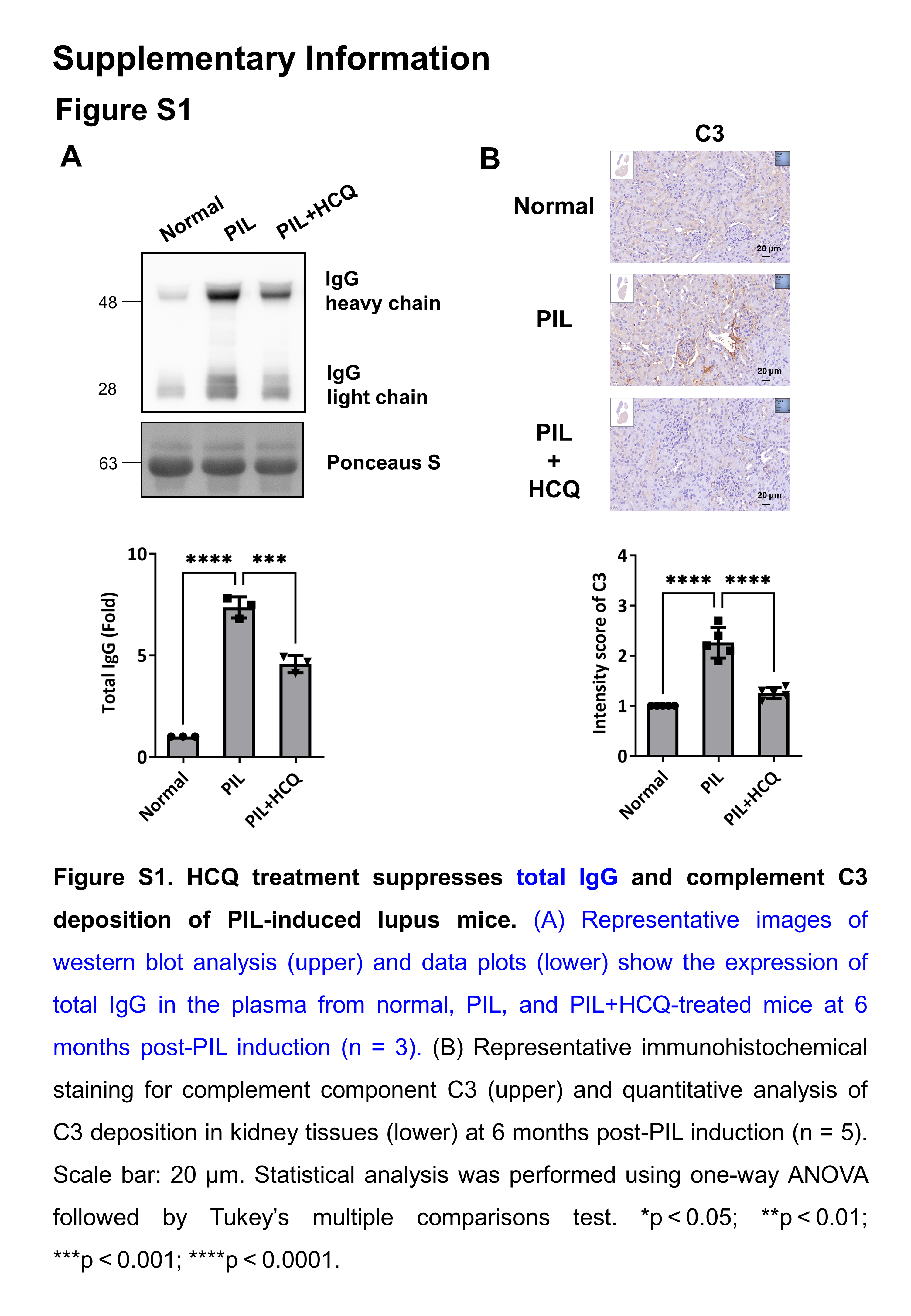

Supplement: Supplementary file 1 [file Image1.tif]

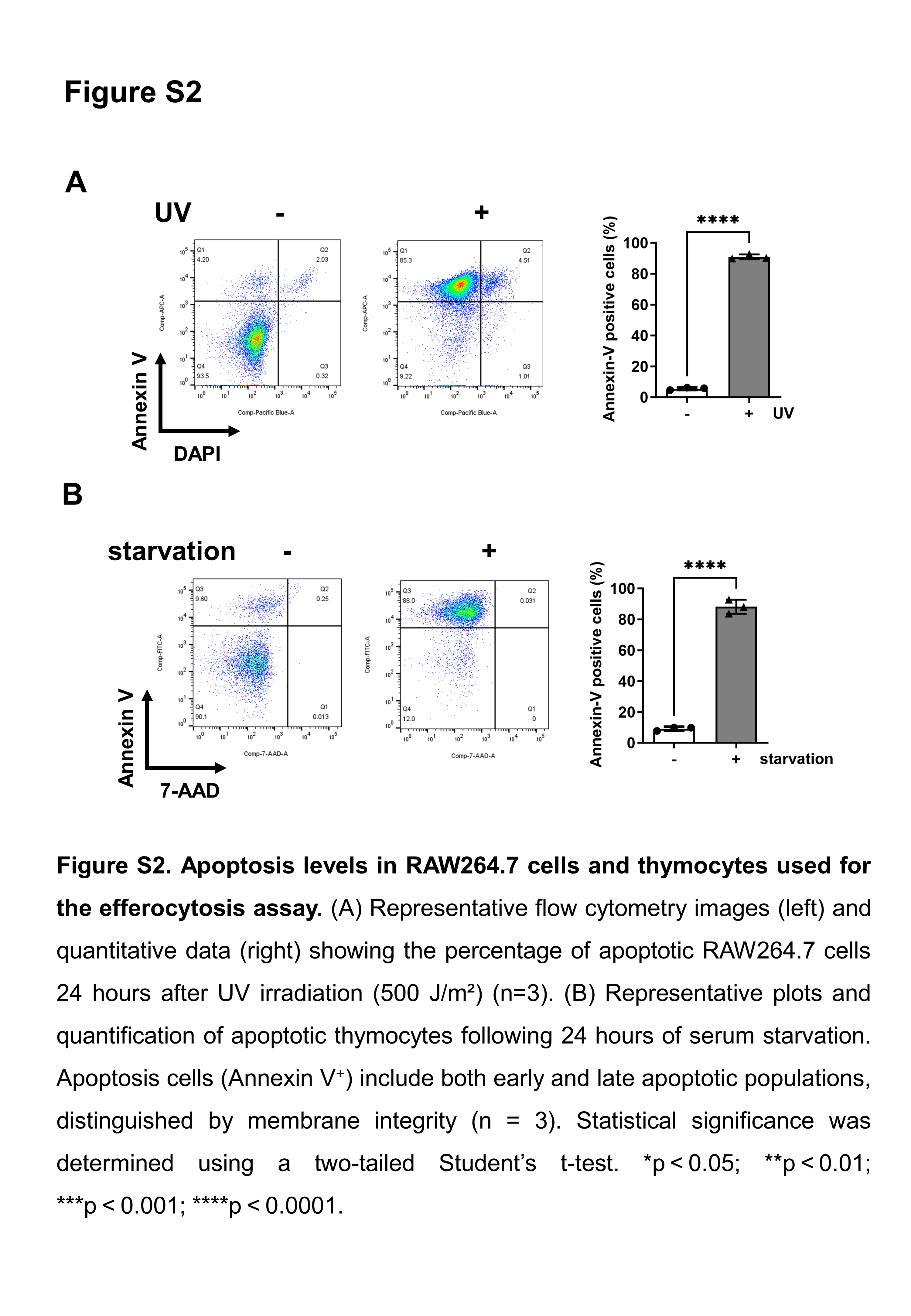

Supplement: Supplementary file 2 [file Image2.tif]

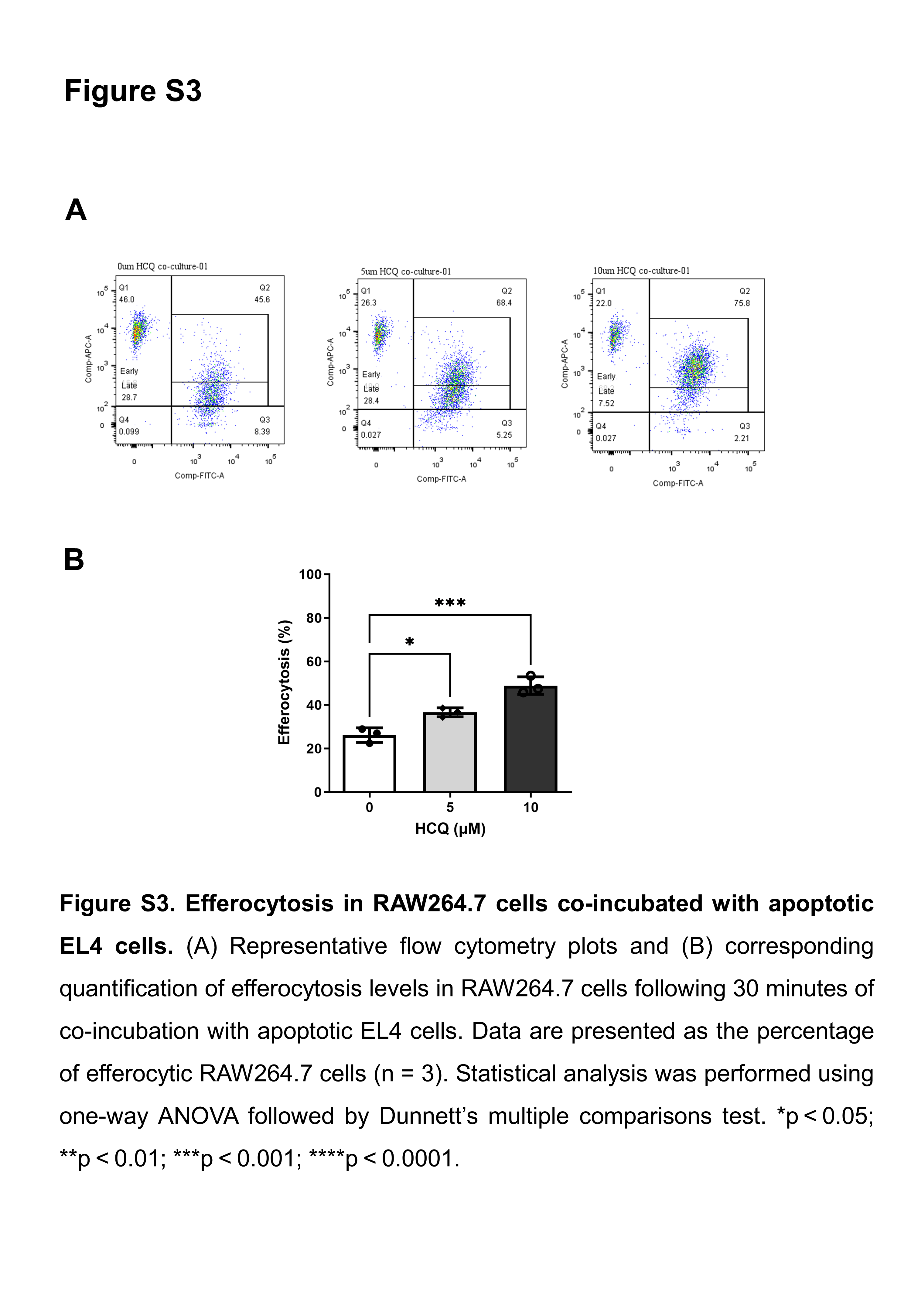

Supplement: Supplementary file 3 [file Image3.tif]

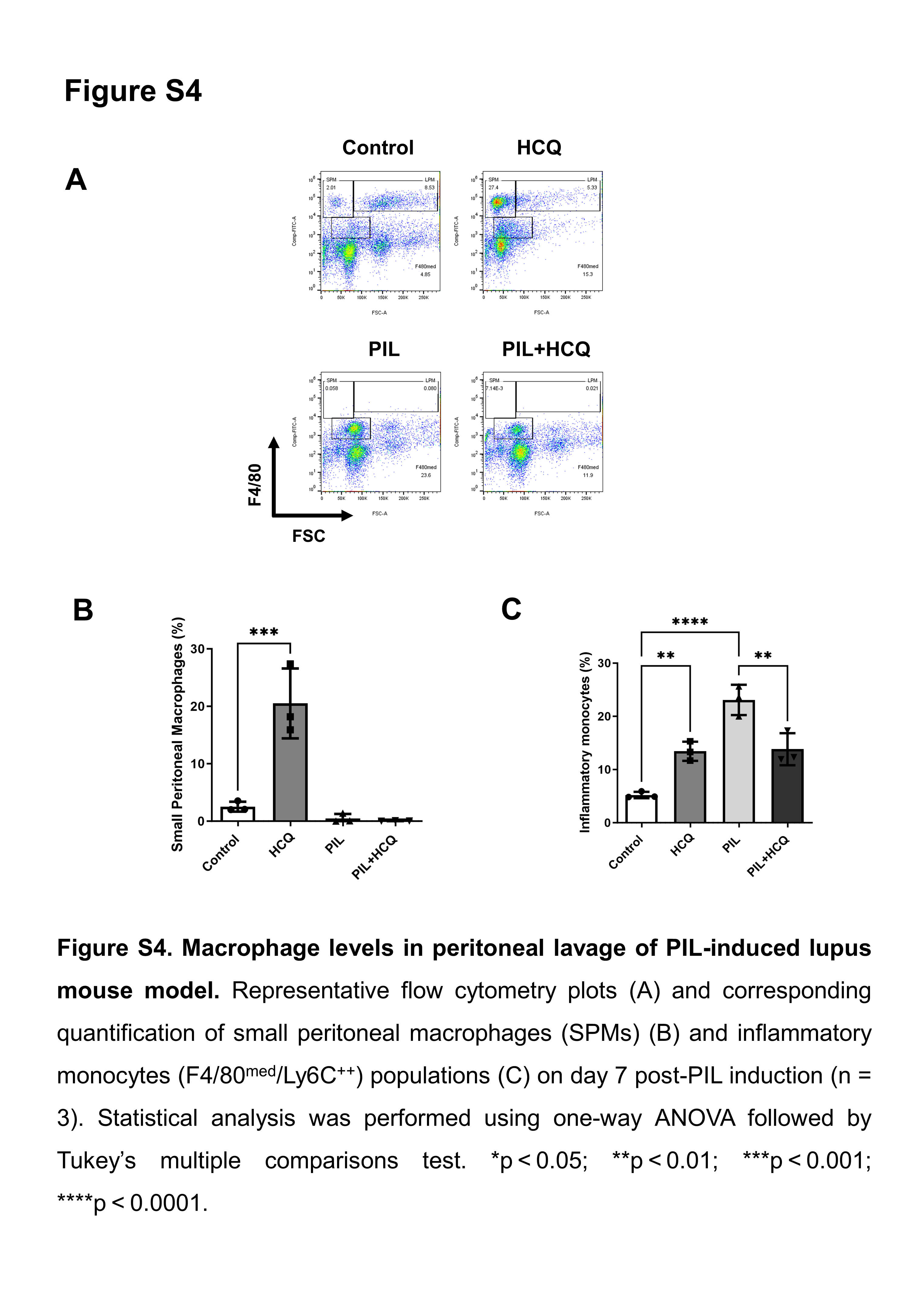

Supplement: Supplementary file 4 [file Image4.tif]

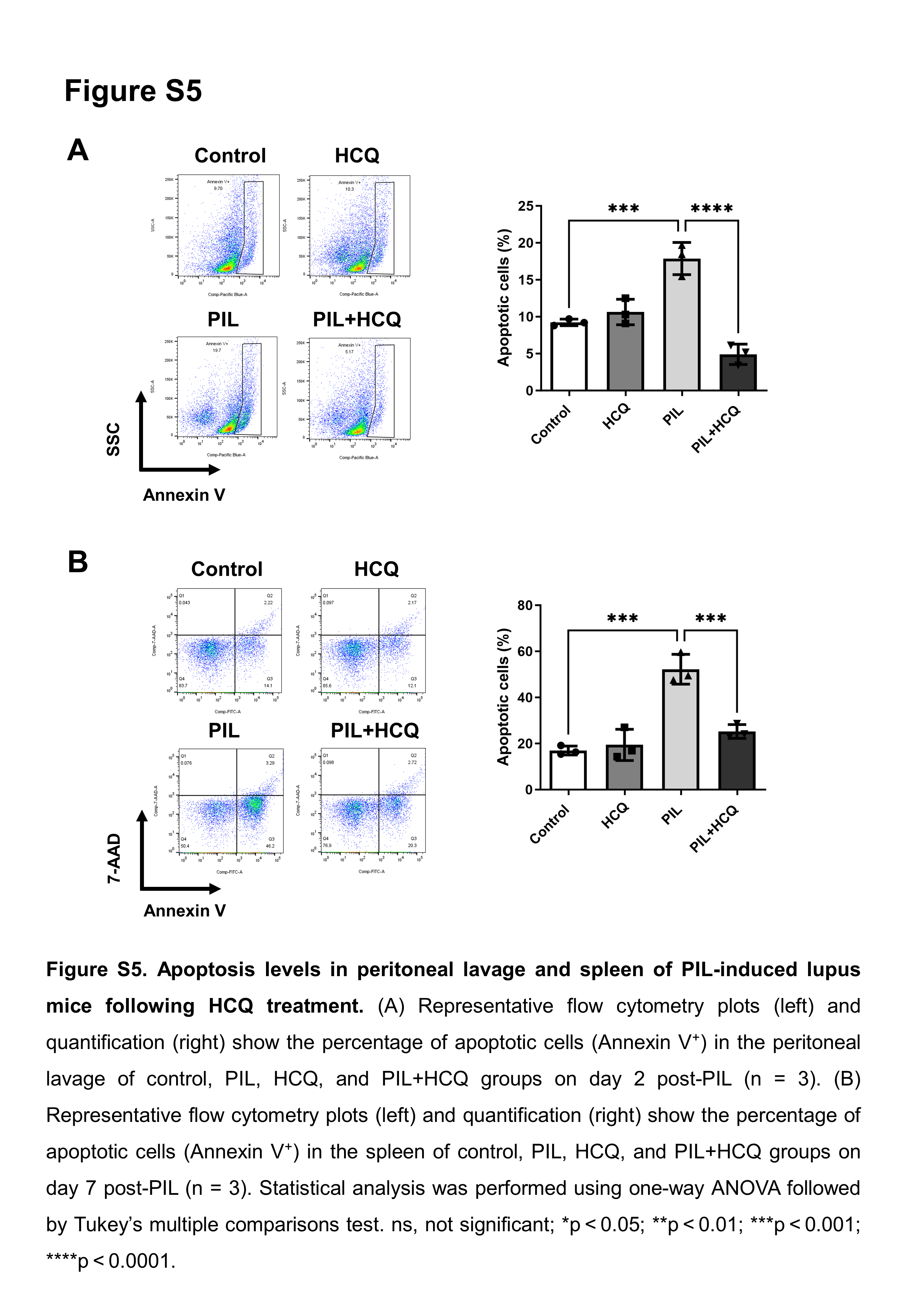

Supplement: Supplementary file 5 [file Image5.tif]

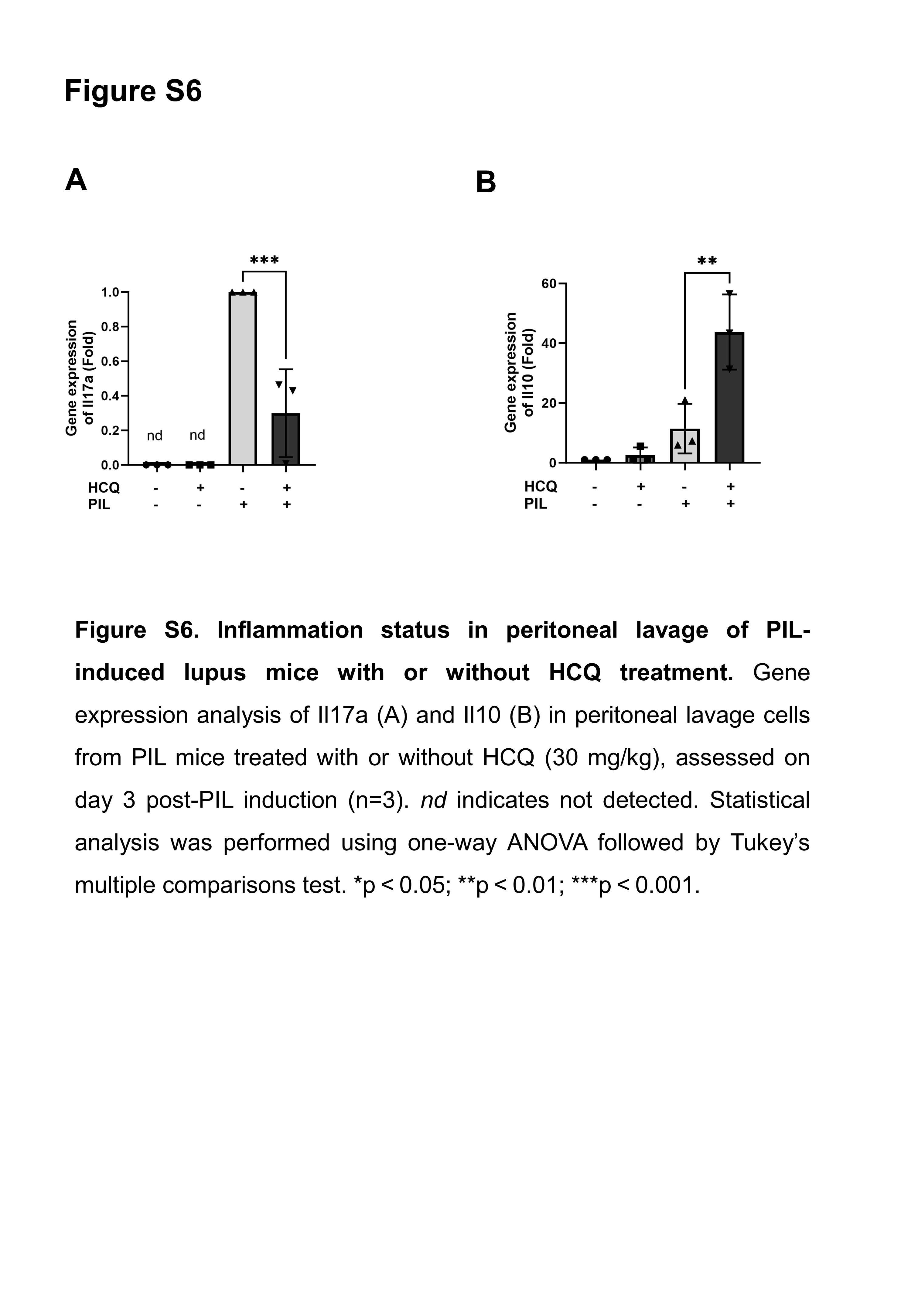

Supplement: Supplementary file 6 [file Image6.tif]

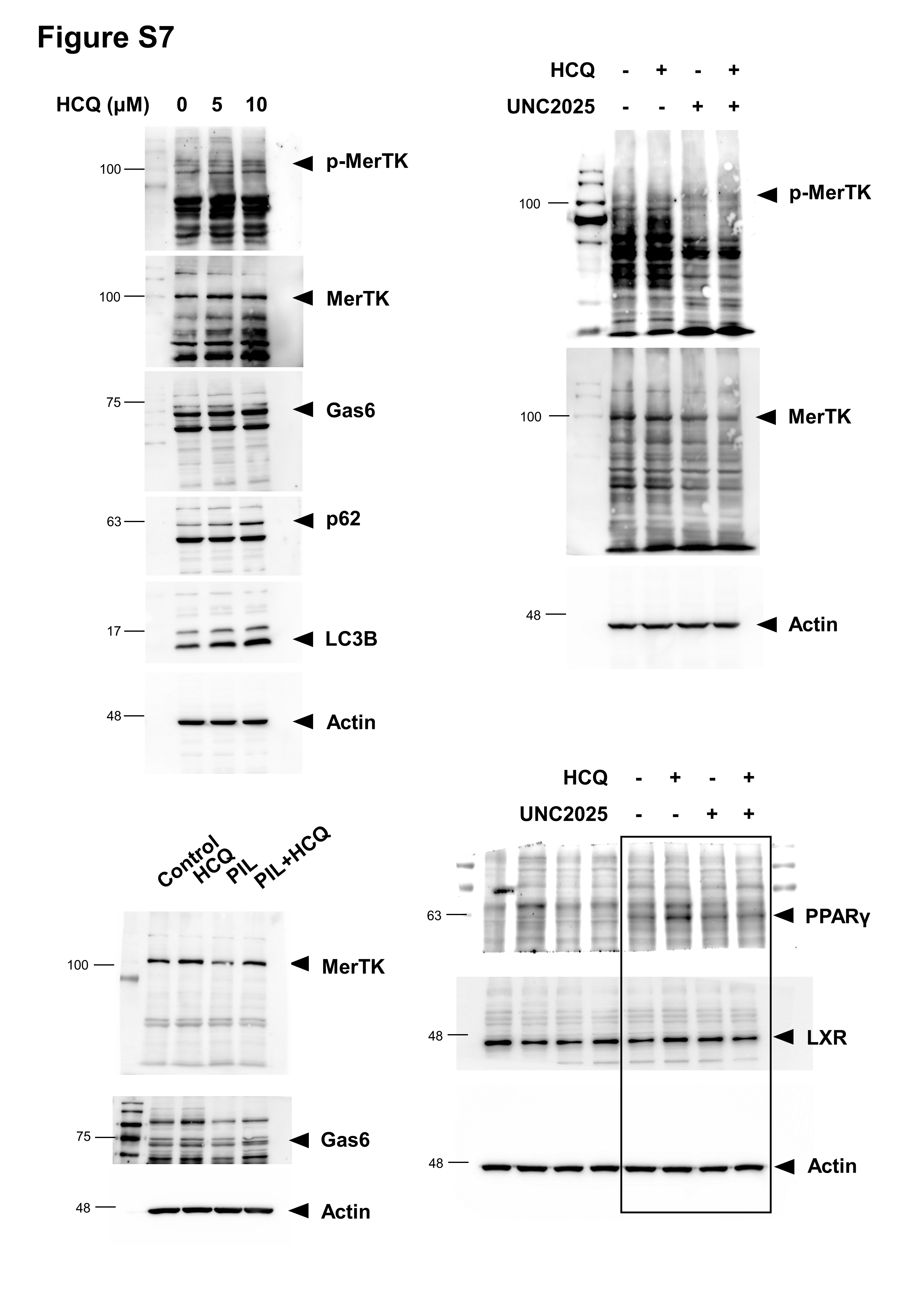

Supplement: Supplementary file 7 [file Image7.tif]
